# Principles of MicroRNA–Target Recognition

**DOI:** 10.1371/journal.pbio.0030085

**Published:** 2005-02-15

**Authors:** Julius Brennecke, Alexander Stark, Robert B Russell, Stephen M Cohen

**Affiliations:** **1**European Molecular Biology LaboratoryHeidelbergGermany

## Abstract

MicroRNAs (miRNAs) are short non-coding RNAs that regulate gene expression in plants and animals. Although their biological importance has become clear, how they recognize and regulate target genes remains less well understood. Here, we systematically evaluate the minimal requirements for functional miRNA–target duplexes in vivo and distinguish classes of target sites with different functional properties. Target sites can be grouped into two broad categories. 5′ dominant sites have sufficient complementarity to the miRNA 5′ end to function with little or no support from pairing to the miRNA 3′ end. Indeed, sites with 3′ pairing below the random noise level are functional given a strong 5′ end. In contrast, 3′ compensatory sites have insufficient 5′ pairing and require strong 3′ pairing for function. We present examples and genome-wide statistical support to show that both classes of sites are used in biologically relevant genes. We provide evidence that an average miRNA has approximately 100 target sites, indicating that miRNAs regulate a large fraction of protein-coding genes and that miRNA 3′ ends are key determinants of target specificity within miRNA families.

## Abstract 2

MicroRNA target site recognition falls into two broad categories: 5' dominant sites that require little support from microRNA 3' end; and 3' compensatory sites that require strong 3' pairing to function

## Introduction

MicroRNAs (miRNAs) are small non-coding RNAs that serve as post-transcriptional regulators of gene expression in plants and animals. They act by binding to complementary sites on target mRNAs to induce cleavage or repression of productive translation (reviewed in [1,2,3,4]). The importance of miRNAs for development is highlighted by the fact that they comprise approximately 1% of genes in animals, and are often highly conserved across a wide range of species (e.g., [5,6,7]). Further, mutations in proteins required for miRNA function or biogenesis impair animal development [8,9,10,11,12,13,14,15].

To date, functions have been assigned to only a few of the hundreds of animal miRNA genes. Mutant phenotypes in nematodes and flies led to the discovery that the *lin-4* and *let-7* miRNAs control developmental timing [16,17], that *lsy-6* miRNA regulates left–right asymmetry in the nervous system [18], that *bantam* miRNA controls tissue growth [19], and that *bantam* and *miR-14* control apoptosis [19,20]. Mouse *miR-181* is preferentially expressed in bone marrow and was shown to be involved in hematopoietic differentiation [21]. Recently, mouse *miR-375* was found to be a pancreatic-islet-specific miRNA that regulates insulin secretion [22].

Prediction of miRNA targets provides an alternative approach to assign biological functions. This has been very effective in plants, where miRNA and target mRNA are often nearly perfectly complementary [23,24,25]. In animals, functional duplexes can be more variable in structure: they contain only short complementary sequence stretches, interrupted by gaps and mismatches. To date, specific rules for functional miRNA–target pairing that capture all known functional targets have not been devised. This has created problems for search strategies, which apply different assumptions about how to best identify functional sites. As a result, the number of predicted targets varies considerably with only limited overlap in the top-ranking targets, indicating that these approaches might only capture subsets of real targets and/or may include a high number of background matches ([19,26,27,28,29,30]; reviewed by [31]). Nonetheless, a number of predicted targets have proven to be functional when subjected to experimental tests [19,26,27,29].

A better understanding of the pairing requirements between miRNA and target would clearly improve predictions of miRNA targets in animals. It is known that defined *cis*-regulatory elements in *Drosophila* 3′ UTRs are complementary to the 5′ ends of certain miRNAs [32]. The importance of the miRNA 5′ end has also emerged from the pairing characteristics and evolutionary conservation of known target sites [26], and from the observation of a non-random statistical signal specific to the 5′ end in genome-wide target predictions [27]. Tissue culture experiments have also underscored the importance of 5′ pairing and have provided some specific insights into the general structural requirements [29,33,34], though different studies have conflicted to some degree with each other, and with known target sites (reviewed in [31]). To date, no specific role has been ascribed to the 3′ end of miRNAs, despite the fact that miRNAs tend to be conserved over their full length.

Here, we systematically evaluate the minimal requirements for a functional miRNA–target duplex in vivo. These experiments have allowed us to identify two broad categories of miRNA target sites. Targets in the first category, “5′ dominant” sites, base-pair well to the 5′ end of the miRNA. Although there is a continuum of 3′ pairing quality within this class, it is useful to distinguish two subtypes: “canonical” sites, which pair well at both the 5′ and 3′ ends, and “seed” sites, which require little or no 3′ pairing support. Targets in the second category, “3′ compensatory” sites, have weak 5′ base-pairing and depend on strong compensatory pairing to the 3′ end of the miRNA. We present evidence that all of these site types are used to mediate regulation by miRNAs and show that the 3′ compensatory class of target sites is used to discriminate among individual members of miRNA families in vivo. A genome-wide statistical analysis allows us to estimate that an average miRNA has approximately 100 evolutionarily conserved target sites, indicating that miRNAs regulate a large fraction of protein-coding genes. Evaluation of 3′ pairing quality suggests that seed sites are the largest group. Sites of this type have been largely overlooked in previous target prediction methods.

## Results

### The Minimal miRNA Target Site

To improve our understanding of the minimal requirements for a functional miRNA target site, we made use of a simple in vivo assay in the *Drosophila* wing imaginal disc. We expressed a miRNA in a stripe of cells in the central region of the disc and assessed its ability to repress the expression of a ubiquitously transcribed enhanced green fluorescent protein (EGFP) transgene containing a single target site in its 3′ UTR. The degree of repression was evaluated by comparing EGFP levels in miRNA-expressing and adjacent non-expressing cells. Expression of the miRNA strongly reduced EGFP expression from transgenes containing a single functional target site (Figure 1A).

**Figure 1:**
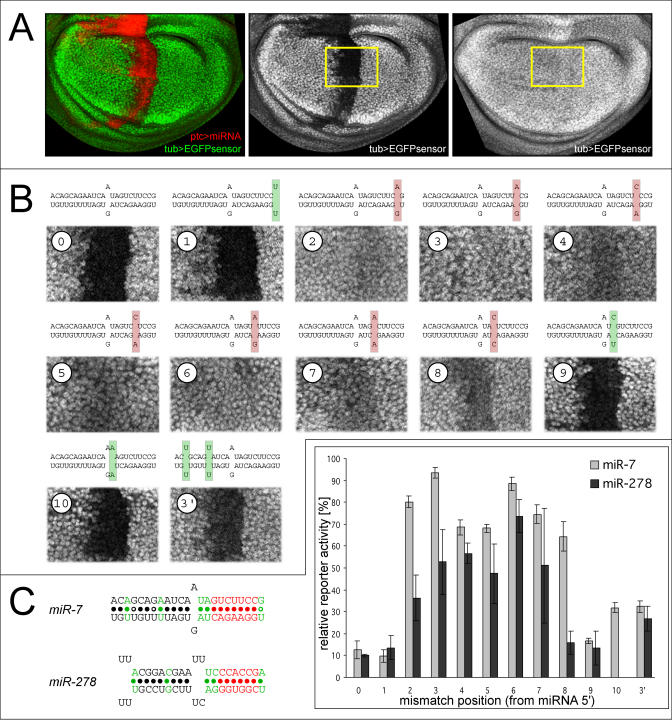
Complementarity to the miRNA 5′ End Is Important for Target Site Function In Vivo (A) In vivo assay for target site regulation in the wing imaginal disc. The EGFP reporter is expressed in all cells (green). Cells expressing the miRNA under ptcGal4 control are shown in red. Functional target sites allow strong GFP repression by the miRNA (middle). Non-functional target sites do not (right). Yellow boxes indicate the disc region shown in (B) and later figures. (B) Regulation of individual target sites by *miR-7.* Numbers in the upper left of each image indicate the mismatched nucleotide in the target site. Positions important for regulation are shown in red, dispensable positions in green. Regulation by the miRNA is completely abolished in only a few cases. (C) Summary of the magnitude of reporter gene repression for the series in (B) and for a second set involving *miR-278* and a target site resembling the *miR-9* site in *Lyra* [26]. Positions important for regulation are shown in red, dispensable positions in green. Error bars are based on measurements of 3–5 individual discs.

In a first series of experiments we asked which part of the RNA duplex is most important for target regulation. A set of transgenic flies was prepared, each of which contained a different target site for *miR-7* in the 3′ UTR of the EGFP reporter construct. The starting site resembled the strongest *bantam* miRNA site in its biological target *hid* [19] and conferred strong regulation when present in a single copy in the 3′ UTR of the reporter gene (Figure 1B). We tested the effects of introducing single nucleotide changes in the target site to produce mismatches at different positions in the duplex with the miRNA (note that the target site mismatches were the only variable in these experiments). The efficient repression mediated by the starting site was not affected by a mismatch at positions 1, 9, or 10, but any mismatch in positions 2 to 8 strongly reduced the magnitude of target regulation. Two simultaneous mismatches introduced into the 3′ region had only a small effect on target repression, increasing reporter activity from 10% to 30%. To exclude the possibility that these findings were specific for the tested miRNA sequence or duplex structure, we repeated the experiment with *miR-278* and a different duplex structure. The results were similar, except that pairing of position 8 was not important for regulation in this case (Figure 1C). Moreover, some of the mismatches in positions 2–7 still allowed repression of EGFP expression up to 50%. Taken together, these observations support previous suggestions that extensive base-pairing to the 5′ end of the miRNA is important for target site function [26,27,29,32,34].

We next determined the minimal 5′ sequence complementarity necessary to confer target regulation. We refer to the core of 5′ sequence complementarity essential for target site recognition as the “seed” (Lewis et al. [27]). All possible 6mer, 5mer, and 4mer seeds complementary to the first eight nucleotides of the miRNA were tested in the context of a site that allowed strong base-pairing to the 3′ end of the miRNA (Figure 2A). The seed was separated from a region of complete 3′ end pairing by a constant central bulge. 5mer and 6mer seeds beginning at positions 1 or 2 were functional. Surprisingly, as few as four base-pairs in positions 2–5 conferred efficient target regulation under these conditions, whereas bases 1–4 were completely ineffective. 4mer, 5mer, or 6mer seeds beginning at position 3 were less effective. These results suggest that a functional seed requires a continuous helix of at least 4 or 5 nucleotides and that there is some position dependence to the pairing, since sites that produce comparable pairing energies differ in their ability to function. For example, the first two duplexes in Figure 2A (4mer, top row) have identical 5′ pairing energies (ΔG for the first 8 nt was −8.9 kcal/mol), but only one is functional. Similarly, the third 4mer duplex and fourth 5mer duplex (middle row) have the same energy (−8.7 kcal/mol), but only one is functional. We thus do not find a clear correlation between 5′ pairing energy and function, as reported in [34]. These experiments also indicate that extensive 3′ pairing of up to 17 nucleotides in the absence of the minimal 5′ element is not sufficient to confer regulation. Consequently, target searches based primarily on optimizing the extent of base-pairing or the total free energy of duplex formation will include many non-functional target sites [28,30,35], and ranking miRNA target sites according to overall complementarity or free energy of duplex formation might not reflect their biological activity [26,27,28,30,35].

**Figure 2:**
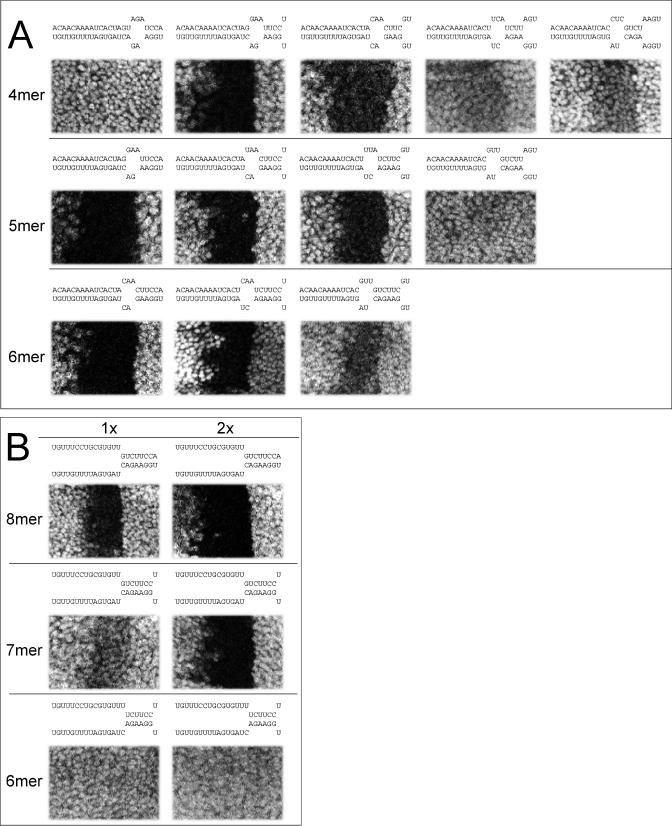
The Minimal miRNA Target Site (A) In vivo tests of the function of target sites with 6mer, 5mer, and 4mer seeds complementary to the first eight nucleotides of the miRNA. Sites were designed to have optimal support from 3′ pairing. The first 4mer seed site shows that extensive complementarity to the miRNA 3′ region is not sufficient for regulation in vivo. (B) Regulation of 8mer, 7mer, and 6mer seed sites lacking complementarity to the miRNA 3′ end. The test UTR contained one site (first column) or two identical sites (second column).

To determine the minimal lengths of 5′ seed matches that are sufficient to confer regulation alone, we tested single sites that pair with eight, seven, or six consecutive bases to the miRNA's 5′ end, but that do not pair to its 3′ end (Figure 2B). Surprisingly, a single 8mer seed (miRNA positions 1–8) was sufficient to confer strong regulation by the miRNA. A single 7mer seed (positions 2–8) was also functional, although less effective. The magnitude of regulation for 8mer and 7mer seeds was strongly increased when two copies of the site were introduced in the UTR. In contrast, 6mer seeds showed no regulation, even when present in two copies. Comparable results were recently reported for two copies of an 8mer site with limited 3′ pairing capacity in a cell-based assay [34]. These results do not support a requirement for a central bulge, as suggested previously [29].

We took care in designing the miRNA 3′ ends to exclude any 3′ pairing to nearby sequence according to RNA secondary structure prediction. However, we cannot rule out the possibility that extensive looping of the UTR sequence might allow the 3′ end to pair to sequences further downstream in our reporter constructs. Note, however, that even if remote 3′ pairing was occurring and required for function of 8- and 7mer seeds, it is not sufficient for 5′ matches with less than seven complementary bases (all test sites are in the same sequence context; Figure 2B). In addition, pairing at a random level will occur in any sequence if long enough loops are allowed. However, whether the ribonucleoprotein complexes involved in translational repression require 3′ pairing, and whether they are able to allow extensive looping to achieve this, remains an open question. Computationally, remote 3′ pairing cannot be distinguished from random matches if loops of any length are allowed. On this basis any site with a 7- or 8mer seed has to be taken seriously—especially when evolutionarily conserved.

From these experiments we conclude that (1) complementarity of seven or more bases to the 5′ end miRNA is sufficient to confer regulation, even if the target 3′ UTR contains only a single site; (2) sites with weaker 5′ complementarity require compensatory pairing to the 3′ end of the miRNA in order to confer regulation; and (3) extensive pairing to the 3′ end of the miRNA is not sufficient to confer regulation on its own without a minimal element of 5′ complementarity.

### The Effect of G:U Base-Pairs and Bulges in the Seed

Several confirmed miRNA target genes contain predicted binding sites with seeds that are interrupted by G:U base-pairs or single nucleotide bulges [17,19,26,36,37,38,39]. In most cases these mRNAs contain multiple predicted target sites and the contributions of individual sites have not been tested. In vitro tests have shown that sites containing G:U base-pairs can function [29,34], but that G:U base-pairs contribute less to target site function than would be expected from their contribution to the predicted base-pairing energy [34]. We tested the ability of single sites with seeds containing G:U base-pairs and bulges to function in vivo. One, two, or three G:U base-pairs were introduced into single target sites with 8mer, 7mer, or 6mer seeds (Figure 3A). A single G:U base-pair caused a clear reduction in the efficiency of regulation by an 8mer seed site and by a 7mer seed site. The site with a 6mer seed lost its activity almost completely. Having more than one G:U base-pair compromised the activity of all the sites. As the target sites were designed to allow optimal 3′ pairing, we conclude that G:U base-pairs in the seed region are always detrimental.

**Figure 3:**
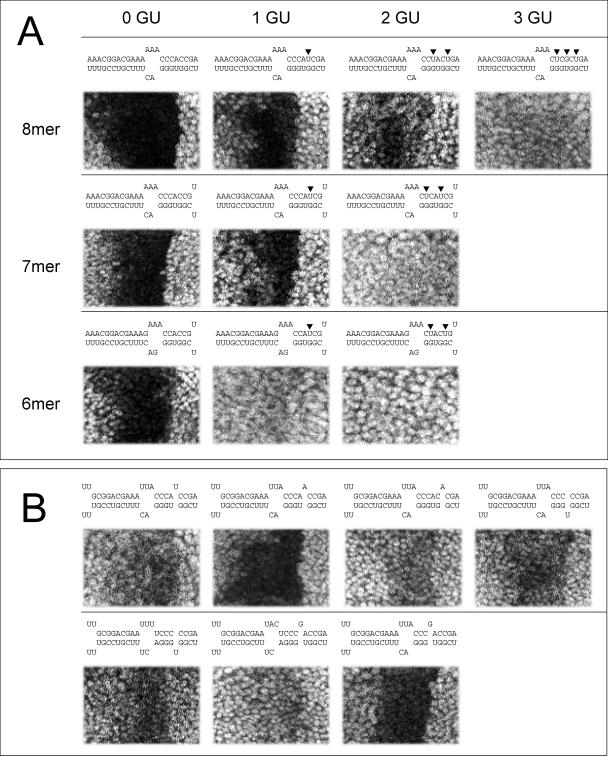
Effects of G:U Base-Pairs and Bulges (A) Regulation of sites with 8mer, 7mer, or 6mer seeds (rows) containing zero, one, two, or three G:U base-pairs in the seed region (columns). (B) Regulation of sites with bulges in the target sequence or in the miRNA.

Single nucleotide bulges in the seed are found in the *let-7* target *lin-41* and in the *lin-4* target *lin-14* [17,36,37]. Recent tissue culture experiments have led to the proposal that such bulges are tolerated if positioned symmetrically in the seed region [29]. We tested a series of sites with single nucleotide bulges in the target or the miRNA (Figure 3B). Only some of these sites conferred good regulation of the reporter gene. Our results do not support the idea that such sites depend on a symmetrical arrangement of base-pairs flanking the bulge. We also note that the identity of the bulged nucleotide seems to matter. While it is clear that some target sites with one nucleotide bulge or a single mismatch can be functional if supported by extensive complementarity to the miRNA 3′ end, it is not possible to generalize about their potential function.

### Functional Categories of Target Sites

While recognizing that there is a continuum of base-pairing quality between miRNAs and target sites, the experiments presented above suggest that sites that depend critically on pairing to the miRNA 5′ end (5′ dominant sites) can be distinguished from those that cannot function without strong pairing to the miRNA 3′ end (3′ compensatory sites). The 3′ compensatory group includes seed matches of four to six base-pairs and seeds of seven or eight bases that contain G:U base-pairs, single nucleotide bulges, or mismatches.

We consider it useful to distinguish two subgroups of 5′ dominant sites: those with good pairing to both 5′ and 3′ ends of the miRNA (canonical sites) and those with good 5′ pairing but with little or no 3′ pairing (seed sites). We consider seed sites to be those where there is no evidence for pairing of the miRNA 3′ end to nearby sequences that is better than would be expected at random. We cannot exclude the possibility that some sites that we identify as seed sites might be supported by additional long-range 3′ pairing. Computationally, this is always possible if long enough loops in the UTR sequence are allowed. Whether long loops are functional in vivo remains to be determined.

Canonical sites have strong seed matches supported by strong base-pairing to the 3′ end of the miRNA. Canonical sites can thus be seen as an extension of the seed type (with enhanced 3′ pairing in addition to a sufficient 5′ seed) or as an extension of the 3′compensatory type (with improved 5′ seed quality in addition to sufficient 3′ pairing). Individually, canonical sites are likely to be more effective than other site types because of their higher pairing energy, and may function in one copy. Due to their lower pairing energies, seed sites are expected to be more effective when present in more than one copy. Figure 4 presents examples of the different site types in biologically relevant miRNA targets and illustrates their evolutionary conservation in multiple drosophilid genomes.

**Figure 4:**
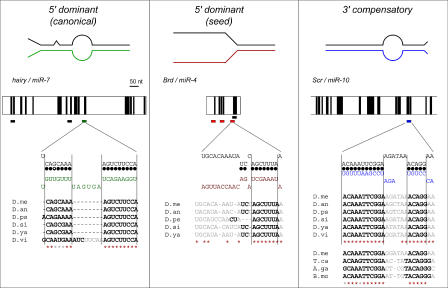
Three Classes of miRNA Target Sites Model of canonical (left), seed (middle) and 3′ compensatory (right) target sites. The upper diagram illustrates the mode of pairing between target site (upper line) and miRNA (lower line, color). Next down in each column are diagrams of the pattern of 3′ UTR conservation. The vertical black bars show stretches of at least six nucleotides that are conserved in several drosophilid genomes. Target sites for *miR-7, miR-4,* and *miR-10* are shown as colored horizontal bars beneath the UTR. Sites for other miRNAs are shown as black bars. Furthest down in each column the predicted structure of the duplex between the miRNA and its target site is shown; canonical base-pairs are marked with filled circles, G:U base-pairs with open circles. The sequence alignments show nucleotide conservation of these target sites in the different drosophilid species Nucleotides predicted to pair to the miRNA are shown in bold; nucleotides predicted to be unpaired are grey. Red asterisks indicate 100% sequence conservation; grey asterisks indicate conservation of base-pairing to the miRNA including G:U pairs. The additional sequence alignment for the *miR-10* target site in *Scr* in *Tribolium castanaeum, Anopheles gambiae,* and Bombyx mori strengthens this prediction. Note that the reduced quality of 3′ compensation in these species is compensated by the presence of a better quality 7mer seed. *A. ga, Anopheles gambiae; B. mo, B. mori; D. an, D. ananassae; D. me, D. melanogaster; D. ps, D. pseudoobscura; D. si, D. simulans; D. vi, D. virilis; D. ya, D. yakuba; T. ca, T. castanaeum.*

Most currently identified miRNA target sites are canonical. For example, the *hairy* 3′ UTR contains a single site for *miR-7,* with a 9mer seed and a stretch of 3′ complementarity. This site has been shown to be functional in vivo [26], and it is strikingly conserved in the seed match and in the extent of complementarity to the 3′ end of *miR-7* in all six orthologous 3′ UTRs.

Although seed sites have not been previously identified as functional miRNA target sites, there is some evidence that they exist in vivo. For example, the *Bearded (Brd)* 3′ UTR contains three sequence elements, known as Brd boxes, that are complementary to the 5′ region of *miR-4* and *miR-79* [32,40]. Brd boxes have been shown to repress expression of a reporter gene in vivo, presumably via miRNAs, as expression of a *Brd* 3′ UTR reporter is elevated in *dicer-1* mutant cells, which are unable to produce any miRNAs [14]. All three Brd box target sites consist of 7mer seeds with little or no base-pairing to the 3′ end of either *miR-4* or *miR-79* (see below). The alignment of *Brd* 3′ UTRs shows that there is little conservation in the *miR-4* or *miR-79* target sites outside the seed sequence, nor is there conservation of pairing to either miRNA 3′ end. This suggests that the sequences that could pair to the 3′ end of the miRNAs are not important for regulation as they do not appear to be under selective pressure. This makes it unlikely that a yet unidentified Brd box miRNA could form a canonical site complex.

The 3′ UTR of the HOX gene *Sex combs reduced (Scr)* provides a good example of a 3′ compensatory site. *Scr* contains a single site for *miR-10* with a 5mer seed and a continuous 11-base-pair complementarity to the miRNA 3′ end [28]. The *miR-10* transcript is encoded within the same HOX cluster downstream of *Scr,* a situation that resembles the relationship between *miR-iab-5p* and *Ultrabithorax* in flies [26] and *miR-196*/*HoxB8* in mice [41]. The predicted pairing between *miR-10* and *Scr* is perfectly conserved in all six drosophilid genomes, with the only sequence differences occurring in the unpaired loop region. The site is also conserved in the 3′ UTR of the Scr genes in the mosquito, Anopheles gambiae, the flour beetle, Tribolium castaneum, and the silk moth,Bombyx mori. Conservation of such a high degree of 3′ complementarity over hundreds of millions of years of evolution suggests that this is likely to be a functional *miR-10* target site. Extensive 5′ and 3′ sequence conservation is also seen for other 3′ compensatory sites, e.g., the two *let-7* sites in *lin-41* or the *miR-2* sites in *grim* and *sickle* [17,26,36].

### The miRNA 3′ End Determines Target Specificity within miRNA Families

Several families of miRNAs have been identified whose members have common 5′ sequences but differ in their 3′ ends. In view of the evidence that 5′ ends of miRNA are functionally important [26,27,29,42], and in some cases sufficient (present study), it can be expected that members of miRNA families may have redundant or partially redundant functions. According to our model, 5′ dominant canonical and seed sites should respond to all members of a given miRNA family, whereas 3′ compensatory sites should differ in their sensitivity to different miRNA family members depending on the degree of 3′ complementarity. We tested this using the wing disc assay with 3′ UTR reporter transgenes and overexpression constructs for various miRNA family members.

*miR-4* and *miR-79* share a common 5′ sequence that is complementary to a single 8mer seed site in the *bagpipe* 3′ UTR (Figure 5A and 5B). The 3′ ends of the miRNAs differ. *miR-4* is predicted to have 3′ pairing at approximately 50% of the maximally possible level (−10.8 kcal/mol), whereas the level of 3′ pairing for *miR-79* is approximately 25% maximum (−6.1 kcal/mol), which is below the average level expected for random matches (see below). Both miRNAs repressed expression of the *bagpipe* 3′ UTR reporter, regardless of the 3′ complementarity (Figure 5B). This indicates that both types of site are functional in vivo and suggests that *bagpipe* is a target for both miRNAs in this family.

**Figure 5:**
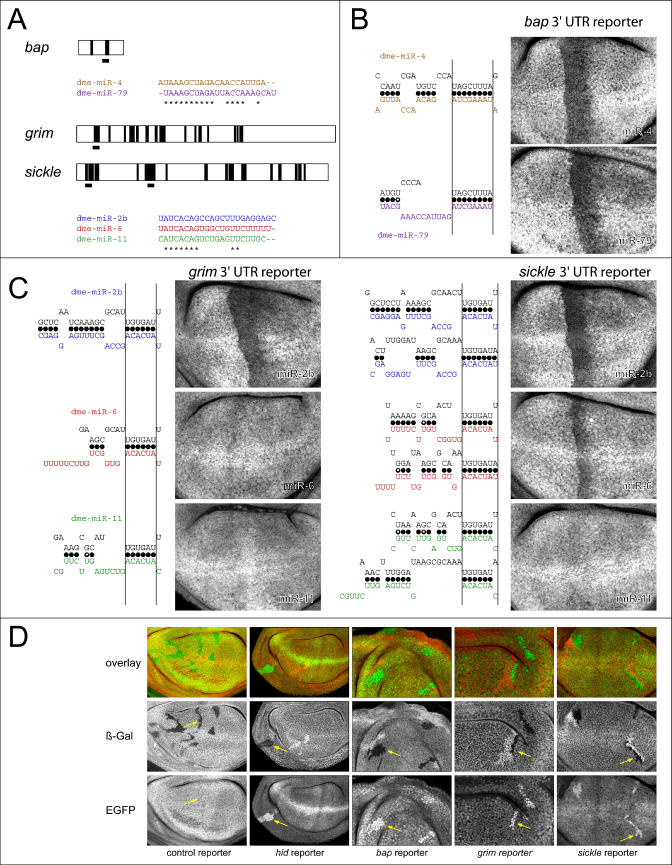
Target Specificity of miRNA Family Members (A) Diagrams of 3′ UTR conservation in six drosophilid genomes (horizontal black bars) and the location of predicted miRNA target sites. Above is the 3′ UTR of the myogenic transcription factor *bagpipe (bap)* showing the predicted target site for the Brd box miRNA family, *miR-4* and *miR-79* (black box below the UTR). Alignment of *miR-4* and *miR-79* illustrates that they share a similar seed sequence (except that *mir-4* has one extra 5′ base) but have little 3′ end similarity. Below are the conserved sequences in the3′ UTRs of the pro-apoptotic genes *grim* and *sickle.* Predicted target sites for the K Box miRNAs *miR-11, miR-2b,* and *miR-6* are shown below the UTR. Alignment of *miR-11, miR-2b,* and *miR-6* illustrates that they share the same family motif but have little similarity in their 3′ ends. (B) The *bagpipe (bap)* 3′ UTR reporter gene is regulated by *miR-4* and *miR-79.* Alignments of the two miRNAs to the predicted target site show good 8mer seed matches (left). Overexpression of *miR-4* or *miR-79* under ptcGal4 control downregulated the *bagpipe* 3′ UTR reporter (right). (C) Left: Alignment of K box miRNAs with the single predicted site in the *grim* 3′ UTR and regulation by overexpression of *miR-2* (top), but not by *miR-6* (middle) or *miR-11* (bottom). Right: Alignment of K box miRNAs with the two predicted sites in the *sickle* 3′ UTR. Regulation by overexpression of *miR-2* was strong (top), regulation by *miR-6* was weaker (middle), and *miR-11* had little effect (bottom). (D) Effect of clones of cells lacking *dicer-1* on expression of UTR reporters for predicted miRNA-regulated genes. Mutant cells were marked by the absence of β-Gal expression (red). EGFP expression is shown in green. Both channels are shown separately below in black and white. Mutant clones are indicated by yellow arrows. Expression of a uniformly transcribed reporter construct lacking miRNA target sites was unaffected in *dicer-1* mutant cells (first column). The UTR reporter for the *bantam* miRNA target *hid* was upregulated in the mutant cells (second column). The *bagpipe (bap)* UTR reporter was upregulated in *dicer-1* clones (third column). The *grim* (fourth column) and *sickle* (fifth column) UTR reporters were upregulated.

To test whether miRNA family members can also have non-overlapping targets, we used 3′ UTR reporters of the pro-apoptotic genes *grim* and *sickle,* two recently identified miRNA targets [26]. Both genes contain K boxes in their 3′ UTRs that are complementary to the 5′ ends of the *miR-2, miR-6,* and *miR-11* miRNA family [26,32]. These miRNAs share residues 2–8 but differ considerably in their 3′ regions (Figure 5A). The site in the *grim* 3′ UTR is predicted to form a 6mer seed match with all three miRNAs (Figure 5C, left), but only *miR-2* shows the extensive 3′ complementarity that we predict would be needed for a 3′ compensatory site with a 6mer seed to function (−19.1 kcal/mol, 63% maximum 3′ pairing, versus −10.9 kcal/mol, 46% maximum, for *miR-11* and −8.7 kcal/mol, 37% maximum, for *miR-6*). Indeed, only *miR-2* was able to regulate the *grim* 3′ UTR reporter, whereas *miR-6* and *miR-11* were non-functional.

The *sickle* 3′ UTR contains two K boxes and provides an opportunity to test whether weak sites can function synergistically. The first site is similar to the *grim* 3′ UTR in that it contains a 6mer seed for all three miRNAs but extensive 3′ complementarity only to *miR-2*. The second site contains a 7mer seed for *miR-2* and *miR-6* but only a 6mer seed for *miR-11* (Figure 5C, right). *miR-2* strongly downregulated the *sickle* reporter, *miR-6* had moderate activity (presumably via the 7mer seed site), and *miR-11* had nearly no activity, even though the miRNAs were overexpressed. The fact that a site is targeted by at least one miRNA argues that it is accessible (e.g., *miR-2* is able to regulate both UTR reporters), and that the absence of regulation for other family members is due to the duplex structure. These results are in line with what we would expect based on the predicted functionality of the individual sites, and indicate that our model of target site functionality can be extended to UTRs with multiple sites. Weak sites that do not function alone also do not function when they are combined.

To show that endogenous miRNA levels regulate all three 3′ UTR reporters, we compared EGFP expression in wild-type cells and *dicer-1* mutant cells, which are unable to produce miRNAs [14]. *dicer-1* clones did not affect a control reporter lacking miRNA binding sites, but showed elevated expression of a reporter containing the 3′ UTR of the previously identified *bantam* miRNA target *hid* (Figure 5D). Similarly, all 3′ UTR reporters above were upregulated in *dicer-1* mutant cells, indicating that *bagpipe, sickle,* and *grim* are subject to repression by miRNAs expressed in the wing disc. Taken together, these experiments indicate that transcripts with 5′ dominant canonical and seed sites are likely to be regulated by all members of a miRNA family. However, transcripts with 3′ compensatory sites can discriminate between miRNA family members.

### Genome-Wide Occurrence of Target Sites

Experimental tests such as those presented above and the observed evolutionary conservation suggest that all three types of target sites are likely to be used in vivo. To gain additional evidence we examined the occurrence of each site type in all Drosophila melanogaster 3′ UTRs. We made use of the D. pseudoobscura genome, the second assembled drosophilid genome, to determine the degree of site conservation for the three different site classes in an alignment of orthologous 3′ UTRs. From the 78 known *Drosophila* miRNAs, we selected a set of 49 miRNAs with non-redundant 5′ sequences. We first investigated whether sequences complementary to the miRNA 5′ ends were better conserved than would be expected for random sequences. For each miRNA, we constructed a cohort of ten randomly shuffled variants. To avoid a bias for the number of possible target matches, the shuffled variants were required to produce a number of sequence matches comparable (±15%) to the original miRNAs for D. melanogaster 3′ UTRs. 7mer and 8mer seeds complementary to real miRNA 5′ ends were significantly better conserved than those complementary to the shuffled variants. This is consistent with the findings of Lewis et al. [27] but was obtained without the need to use a rank and energy cutoff applied to the full-length miRNA target duplex, as was the case for vertebrate miRNAs. Conserved 8mer seeds for real miRNAs occur on average 2.8 times as often as seeds complementary to the shuffled miRNAs (Figure 6A). For 7mer seeds this signal was 2:1, whereas 6mer, 5mer, and 4mer seeds did not show better conservation than expected for random sequences. To assess the validity of these signals and to control for the random shuffling of miRNAs, we repeated this procedure with “mutant” miRNAs in which two residues in the 5′ region were changed. There was no difference between the mutant test miRNAs and their shuffled variants (Figure 6A). This indicates that a substantial fraction of the conserved 7mer and 8mer seeds complementary to real miRNAs identify biologically relevant target sites.

**Figure 6:**
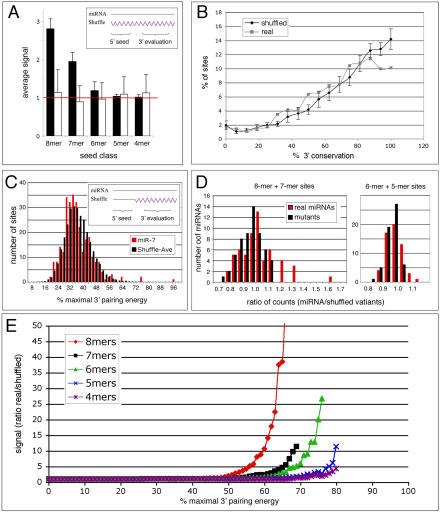
Computational Analysis of Target Site Occurrence (A) Genome-wide occurrence of conserved 5′ seed matches. Histogram showing the ratio of 5′ seed matches for a set of 49 5′ non-redundant miRNAs and the average of their ten completely shuffled variants for different seed types (black bars). A ratio of one (red line) indicates no difference between the miRNA and its shuffled variants. The same ratio for mutated miRNAs and their shuffled variants shows no signal (white bars). The inset depicts shuffling of the entire miRNA sequence (wavy purple line). (B) Target site conservation between D. melanogaster and D. pseudoobscura. Histogram showing the average conservation of the 3′ UTR sequence (16 nt) upstream of a conserved 8mer seed match that would pair to the miRNA 3′ end. All sites were binned according to their conservation, and the percentage of sites in each bin is shown for sites identified by 49 5′ non-redundant miRNA sequences (grey) and their shuffled control sequences (black, error bars indicate one standard deviation). (C) 3′ pairing preferences for *miR-7* target sites. Histogram showing the distribution of 3′ pairing energies for *miR-7* (red bars) and the average of 50 3′ shuffled variants (black bars) for all sites identified genome-wide by 6mer 5′ seed matches for *miR-7.* The inset illustrates shuffling of the 3′ end of miRNA sequence only (wavy purple line). Because the miRNA 5′ end was not altered, the identical set of target sites was compared for pairing to the 3′ end of real and shuffled miRNAs. (D) 3′ pairing preferences for miRNA target sites. Histograms showing the ratio of the top 1% 3′ pairing energies for the set of 58 3′ non-redundant miRNAs and their shuffled variants. The y-axis shows the number of miRNAs for each ratio. Real miRNAs are shown in red; mutant miRNAs are shown in black. Left are shown combined 8- and 7mer seed sites. Right are shown combined 5- and 6mer seed sites. For combined 8- and 7mer seeds, 1% corresponds to approximately ten sites per miRNA; for combined 6- and 5mer, to approximately 25 sites. The difference between the real and mutated miRNAs improves if fewer sites per miRNA are considered. (E) Non-random signal of 3′ pairing. Plot of the ratio of the number of target sites for the set of 58 3′ non-redundant miRNAs and their shuffled miRNA 3′ ends (y-axis) with 3′ pairing energies that exceed a given pairing cutoff (x-axis). 100% is the pairing energy for a sequence perfectly complementary to the 3′ end. As the required level of 3′ pairing energy increases, fewer miRNAs and their sites remain to contribute to the signal. Plots for the real miRNAs extended to considerably higher 3′ pairing energies than the mutants, but as site number decreases we observe anomalous effects on the ratios, so the curves were cut off when the number of remaining miRNAs fell below five.

3′ compensatory and canonical sites depend on substantial pairing to the miRNA 3′ end. For these sites, we expect UTR sequences adjacent to miRNA 5′ seed matches to pair better to the miRNA 3′ end than to random sequences. However, unlike 5′ complementarity, 3′ base-pairing preference was not detected in previous studies looking at sequence complementarity and nucleotide conservation because UTR sequences complementary to the miRNA 3′ end were not better conserved than would be expected at random [27].

On this basis, we decided to treat the 5′ and 3′ ends of the miRNA separately. For the 5′ end, seed matches were required to be fully conserved in an alignment of orthologous D. melanogaster and D. pseudoobscura 3′ UTRs (we expected one-half to two-thirds of these matches to be real miRNA sites). We first investigated the overall conservation of UTR sequences adjacent to the conserved seed matches and found that overall the sequences are not better conserved than a random control with shuffled miRNAs (Figure 6B). For both real and random matches, the number of sites increases with the degree of 3′ conservation (up to the 80% level), reflecting the increased probability that sequences adjacent to conserved seed matches will also lie in blocks of conserved sequence (Figure 6B). For real 7mers and 8mers we found a slightly higher percentage of sites between 30% and 80% identity than we did for the shuffled controls. In contrast, the ratio of sites with over 80% sequence identity was smaller for real 7- or 8mers than for random ones, meaning that in highly conserved 3′ UTR blocks (>80% identity) the ratio of random matches exceeds that of real miRNA target sites. This caused us to question whether the degree of conservation for sequences adjacent to seed matches correlates with miRNA 3′ pairing as would be expected if the conservation were due to a biologically relevant miRNA target site. Indeed, we found that the best conserved sites adjacent to seed matches (i.e., those with zero, one, or two mismatches in the 3′ UTR alignment) and the least conserved sites (i.e., those with only three, two, or one matching nucleotides) are not distinguishable in that both pair only randomly to the corresponding miRNA 3′ end (approximately 35% maximal 3′ pairing energy, data not shown). The observation that miRNA target sites do not seem to be fully conserved over their entire length is consistent with the examples shown in Figure 4 in which only the degree of 3′ pairing but not the nucleotide identity is conserved *(miR-7/hairy),* or at least the unpaired bulge is apparently not under evolutionary pressure *(miR-10/Scr).* Although this result obviously depends on the evolutionary distance of the species under consideration (see [43] for a comparison of mammalian sites), it shows that conclusions about the contribution of miRNA 3′ pairing to target site function cannot be drawn solely from the degree of sequence conservation.

We therefore chose to evaluate the quality of 3′ pairing by the stability of the predicted RNA–RNA duplex. We assessed predicted pairing energy between the miRNA 3′ end and the adjacent UTR sequence for both *Drosophila* species and used the lower score. Use of the lower score measures conservation of the overall degree of pairing without requiring sequence identity. Figure 6C shows the distribution of the 3′ pairing energies for all conserved 3′ compensatory *miR-7* sites identified by a 6mer seed match, compared to the distribution of 50 *miR-7* sequences shuffled only in the 3′ part, leaving the 5′ unchanged. This means that real and shuffled miRNAs identify the same 5′ seed matches in the 3′ UTRs, which allows us to compare the 3′ pairing characteristics of the adjacent sequences. We also required 3′ shuffled sequences to have similar pairing energies (±15%) to their complementary sequences and to 10,000 randomly selected sites to exclude generally altered pairing characteristics. The distributions for real and shuffled miRNAs were highly similar, with a mean of approximately 35% of maximal 3′ pairing energy and few sites above 55%. However, a small number of sites paired exceptionally well to *miR-7* at energies that were far above the shuffled averages and not reached by any of the 50 shuffled controls. This example illustrates that there is a significant difference between real and shuffled miRNAs for the sites with the highest 3′ complementarity, which are likely to be biologically relevant. Sites with weaker 3′ pairing might also be functional, but cannot be distinguished from random matches and can only be validated by experiments (see Figure 5). To provide a global analysis of 3′ pairing comprising all miRNAs and to investigate how many miRNAs show significantly non-random 3′ pairing, we considered only the sites within the highest 1% of 3′ pairing energies.

The average of the highest 1% of 3′ pairing energies of each of 58 3′ non-redundant miRNAs was divided by that of its 50 3′ shuffled controls. This ratio is one if the averages are the same, and increases if the real miRNA has better 3′ pairing than the shuffled miRNAs. To test whether a signal was specific for real miRNAs, we repeated the same protocol with a mutant version of each miRNA. The altered 5′ sequence in the mutant miRNA selects different seed matches than the real miRNA and permits a comparison of sequences that have not been under selection for complementarity to miRNA 3′ ends with those that may have been. Figure 6D shows the distribution of the energy ratios for canonical (left) and 3′ compensatory sites (right) for all 58 real and mutated 3′ non-redundant miRNAs. Most real miRNAs had ratios close to one, comparable to the mutants. But several had ratios well above those observed for mutant miRNAs, indicating significant conserved 3′ pairing.

A small fraction of sites show exceptionally good 3′ pairing. If we use 3′ pairing energy cutoffs to examine site quality for all miRNAs, we expect sites of this type to be distinguishable from random matches. The ratio of the number of sites above the cutoff for real versus 3′ shuffled miRNAs was plotted as a function of the 3′ pairing cutoff (Figure 6E). For low cutoffs the ratio is one, as the number of sites corresponds to the number of seed matches (which is identical for real and 3′ shuffled miRNAs). For increasing cutoffs, the ratios increase once a certain threshold is reached, reflecting overrepresentation of sites that pair favorably to the real miRNA 3′ end but not the 3′ shuffled miRNAs. The maximal ratio obtained for mutated miRNAs never exceeded five, which we used as the threshold level to define where significant overrepresentation begins. For 8mer seed sites overrepresentation began at 55% maximal 3′ pairing; for 7mer seed sites, at 65%; for 6mer seed sites, at 68%; and for 5mer seed sites, at 78%. There was no statistical evidence for sites with 4mer seeds.

We also tested whether sequences forming 7mer or 8mer seeds containing G:U base-pairs, mismatches, or bulges were better conserved if complementary to real miRNAs. We did not find any statistical evidence for these seed types. Analysis of 3′ pairing also failed to show any non-random signal for these sites. This suggests that such sites are few in number genome-wide and are not readily distinguished from random matches. Nonetheless, our experiments do show that sites of this type can function in vivo. The *let-7* sites in *lin-41* provide a natural example.

### Most Sites Lack Substantial 3′ Pairing

The experimental and computational results presented above provide information about 5′ and 3′ pairing that allows us to estimate the number of target sites of each type in *Drosophila.* The number of 3′ compensatory sites cannot be estimated on the basis of 5′ pairing, because seed matches of four, five, or six bases cannot be distinguished from random matches, reflecting that a large number of randomly conserved and non-functional matches predominate (Figure 6A). Significant 3′ pairing can be distinguished from random matches for 6mer sites above 68% maximal 3′ pairing energy, and above 78% for 5mers (Figure 6E). Using these pairing levels gives an estimate of one 3′ compensatory site on average per miRNA. The experiments in Figure 5 provide an opportunity to assess the contribution of 3′ pairing to the ability of sites with 6mer seeds to function. The 6mer K box site in the *grim* 3′ UTR was regulated by *miR-2* (63% maximal 3′ pairing energy), but not by *miR-11,* which has a predicted 3′ pairing energy of 46%. Similarly, the 6mer seed sites for *miR-11* in the *sickle* 3′ UTR had 3′ pairing energies of approximately 35% and were non-functional. We can use the 63% and 46% levels to provide upper and lower estimates of one and 20 3′ compensatory 6mer sites on average per miRNA. For 5mer sites, the examples in Figure 1 show that sites with 76% and 83% maximal 3′ pairing do not function. At the 80% threshold level, we expect less than one additional site on average per miRNA, suggesting that 3′ compensatory sites with 5mer seeds are rare. The predicted *miR-10* site in *Scr* (see Figure 4) is one of the few sites with a 5mer seed that reaches this threshold (100% maximum 3′ pairing energy; −20 kcal/mol). It is likely that other sites in this group will also prove to be functionally important.

The overrepresentation of conserved 5′ seed matches (see Figure 6A) suggests that approximately two-thirds of sites with 8mer seeds and approximately one-half of the sites with 7mer seeds are biologically relevant. This corresponds to an average of 28 8mers and 53 7mers, for a total of 81 sites per miRNA. We define canonical sites as those with meaningful contributions from both 5′ and 3′ pairing. Given that 7- and 8mer seed matches can function without significant 3′ pairing, it is difficult to assess at what level 3′ pairing contributes meaningfully to their function. The range of 3′ pairing energies that were minimally sufficient to support a weak seed match was between 46% and 63% of maximum pairing energy (see Figure 5C). If we take the 46% level as the lower limit for meaningful 3′ pairing, over 95% of sites would be considered seed sites. This changes to 99% for pairing energies that can be statistically distinguished from noise (55% maximal; see Figure 6E) and remains over 50% even for pairing energies at the average level achieved by random matches (30% maximal). It is clear from this analysis that the majority of miRNA target sites lack substantial pairing in the 3′ end in nearby sequences. Indeed the 3′ pairing level for the three seed sites for *miR-4* in *Brd* are all less than 25% (i.e., below the average for random matches) and *Brd* was thus not predicted as a *miR-4* target previously [26,28,35].

Again, we note the caveat that some of sites that we identify as seed could in principle be supported by 3′ pairing to more distant upstream sequences, but also that such sites would be difficult to distinguish from background computationally and that it is unclear whether large loops are functional. If there were statistical evidence for 3′ pairing that is lower than would be expected at random for some sites, this would be one line of argument for a discrete functional class that does not use 3′ pairing and would therefore suggest selection against 3′ pairing. Although the overall distribution of 3′ pairing energies for real miRNA 3′ ends adjacent to 8mer seed matches is very similar to the random control with 3′ shuffled sequences (Figure 7; *R* ^2^ = 0.98), we observed a small but significant overrepresentation of real sites on both sides of the random distribution, which leads to a slightly wider distribution of real sites at the expense of the peak values around 30% pairing. Bearing in mind that one-third of 8mer seed matches are false positives (see Figure 6A), we can account for the noise by subtracting one-third of the random distribution. We then see two peaks at around 20% and 35% maximum pairing energy, separated by a dip. Subtracting more (e.g., one-half or two-thirds) of the random distribution increases the separation of the two peaks, suggesting that the underlying distribution of 3′ pairing for real 8mer seed sites might indeed be bimodal. This effect is still present, though less pronounced, if 7mer seed matches are included. No such effect is seen for the combined 5- and 6mer seed matches. In addition, we see no difference between a random (noise) model that evaluates 3′ pairing of 3′ shuffled miRNAs to UTR sites identified by real miRNA seed matches and a random model that pairs the real (i.e., non-shuffled) miRNA 3′ end to randomly chosen UTR sequences, thus excluding bias due to shuffling. Overall, these results suggest that there might indeed be a bimodal distribution due to an enrichment of sites with both better and worse 3′ pairing than would be expected at random. We take this as evidence that seed sites are a biologically meaningful subgroup within the 5′ dominant site category.

**Figure 7:**
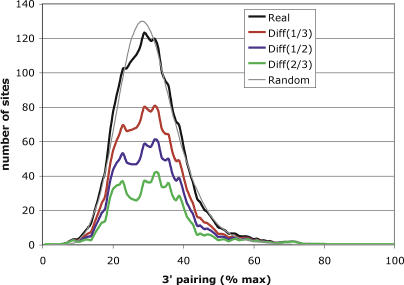
Distribution of 3′ Pairing Energies for 8mer Seed Matches Shown is the distribution (number of sites versus 3′ pairing) for 8mer seed matches identified genome-wide for 58 3′ non-redundant miRNAs (black) compared to a random control using 50 3′ shuffled miRNAs per real miRNA (grey). Note that the distribution for real miRNAs is broader at both the high and low end than the random control and has shoulders close to the peak. The red, blue, and green curves show the effect of subtracting background noise (random matches) from the real matches at three different levels, which reveals the real matches underlying these shoulders.

Overall, these estimates suggest that there are over 80 5′ dominant sites and 20 or fewer 3′ compensatory sites per miRNA in the *Drosophila* genome. As estimates of the number of miRNAs in *Drosophila* range from 96 to 124 [44], this translates to 8,000–12,000 miRNA target sites genome-wide, which is close to the number of protein-coding genes. Even allowing for the fact that some genes have multiple miRNA target sites, these findings suggest that a large fraction of genes are regulated by miRNAs.

## Discussion

We have provided experimental and computational evidence for different types of miRNA target sites. One key finding is that sites with as little as seven base-pairs of complementarity to the miRNA 5′ end are sufficient to confer regulation in vivo and are used in biologically relevant targets. Genome-wide, 5′ dominant sites occur 2- to 3-fold more often in conserved 3′ UTR sequences than would be expected at random. The majority of these sites have been overlooked by previous miRNA target prediction methods because their limited capacity to base-pair to the miRNA 3′ end cannot be distinguished from random noise. Such sites rank low in search methods designed to optimize overall pairing energy [16,17,26,27,28,30,35]. Indeed, we find that few seed sites scored high enough to be considered seriously in these earlier predictions, even when 5′ complementarity was given an additional weighting (e.g., [28,43]. We thus suspect that methods with pairing cutoffs would exclude many, if not all, such sites.

In a scenario in which protein-coding genes acquire miRNA target sites in the course of evolution [4], it is likely that seed sites with only seven or eight bases complementary to a miRNA would be the first functional sites to be acquired. Once present, a site would be retained if it conferred an advantage, and sites with extended complementarity could also be selected to confer stronger repression. In this scenario, the number of sites might grow over the course of evolution so that ancient miRNAs would tend to have more targets than those more recently evolved. Likewise, genes that should not be repressed by the miRNA milieu in a given cell type would tend to avoid seed matches to miRNA 5′ ends (“anti-targets” [4]).

Although a 7- to 8mer seed is sufficient for a site to function, additional 3′ pairing increases miRNA functionality. The activity of a single 7mer canonical site is expected to be greater than an equivalent seed site. Likewise, the magnitude of miRNA-induced repression is reduced by introducing 3′ mismatches into a canonical site. Genome-wide, there are many sites that appear to show selection for conserved 3′ pairing and, interestingly, many sites that appear to show selection against 3′ pairing. In vivo, canonical sites might function at lower miRNA concentrations and might repress translation more effectively, particularly when multiple sites are present in one UTR (e.g., [42]). Efficient repression is likely to be necessary for genes whose expression would be detrimental, as illustrated by the genetically identified miRNAs, which produce clear mutant phenotypes when their targets are not normally repressed (“switch targets” [4]). Prolonged expression of the *lin-14* and *lin-41* genes in Caenorhabditis elegans mutant for *lin-4* or *let-7* causes developmental defects, and their regulation involves multiple sites [17,36,37]. Similarly, multiple target sites allow robust regulation of the pro-apoptotic gene *hid* by *bantam* miRNA in *Drosophila* [19]. More subtle modulation of expression levels could be accomplished by weaker sites, such as those lacking 3′ pairing. Sites that cannot function efficiently alone are in fact a prerequisite for combinatorial regulation by multiple miRNAs. Seed sites might thus be useful for situations in which the combined input of several miRNAs is used to regulate target expression. Depending on the nature of the target sites, any single miRNA might not have a strong effect on its own, while being required in the context of others.

### 3′ Complementarity Distinguishes miRNA Family Members

3′ compensatory sites have weak 5′ pairing and need substantial 3′ pairing to function. We find genome-wide statistical support for 3′ compensatory sites with 5mer and 6mer seeds and show that they are used in vivo. Furthermore, these sites can be differentially regulated by different miRNA family members depending on the quality of their 3′ pairing (e.g., regulation of the pro-apoptotic genes *grim* and *sickle* by *miR-2, miR-6,* and *miR-11*). Thus, members of a miRNA family may have common targets as well as distinct targets. They may be functionally redundant in regulation of some targets but not others, and so we can expect some overlapping phenotypes as well as differences in their mutant phenotypes.

Following this reasoning, it is likely that the *let-7* miRNA family members differentially regulate *lin-41* in C. elegans [17,45]. The seed matches in *lin-41* to *let-7* and the related miRNAs *miR-48, miR-84,* and *miR-241* are weak, and only *let-7* has strong 3′ pairing. On this basis, it seems likely that *lin-41* is regulated only by *let-7*. In contrast, *hbl-1* has four sites with strong seed matches [38,39], and we expect it to be regulated by all four *let-7* family members. As all four *let-7*-related miRNAs are expressed similarly during development [6], their role as regulators of *hbl-1* may be redundant. *let-7* must also have targets not shared by the other family members, as its function is essential. *lin-41* is likely to be one such target.

The idea that the 3′ end of miRNAs serves as a specificity factor provides an attractive explanation for the observation that many miRNAs are conserved over their full length across species separated by several hundreds of millions of years of evolution. 3′ compensatory sites may have evolved from canonical sites by mutations that reduce the quality of the seed match. This could confer an advantage by allowing a site to become differentially regulated by miRNA family members. In addition, sites could retain specificity and overall pairing energy, but with reduced activity, perhaps permitting discrimination between high and low levels of miRNA expression. This might also allow a target gene to acquire a dependence on inputs from multiple miRNAs. These scenarios illustrate a few ways in which more complex regulatory roles for miRNAs might arise during evolution.

### A Large Fraction of the Genome Is Regulated by miRNAs

Another intriguing outcome of this study is evidence for a surprisingly large number of miRNA target sites genome-wide. Even our conservative estimate is far above the numbers of sites in recent predictions, e.g., seven or fewer per miRNA [27,28,29]. Our estimate of the total number of targets approaches the number of protein-coding genes, suggesting that regulation of gene expression by miRNAs plays a greater role in biology than previously anticipated. Indeed, Bartel and Chen [46] have suggested in a recent review that the earlier estimates were likely to be low, and a recent study by John et al. [43], published while this manuscript was under review, predicts that approximately 10% of human genes are regulated by miRNAs. We agree with these authors' suggestion that this is likely an underestimate, because their method identifies an average of only 7.1 target genes per miRNA, with few that we would classify as seed sites lacking substantial 3′ pairing. A large number of target sites per miRNA is also consistent with combinatorial gene regulation by miRNAs, analogous to that by transcription factors, leading to cell-type-specific gene expression [47]. Sites for multiple miRNAs allow for the possibility of cell-type-specific miRNA combinations to confer robust and specific gene regulation.

Our results provide an improved understanding of some of the important parameters that define how miRNAs bind to their target genes. We anticipate that these will be of use in understanding known miRNA–target relationships and in improving methods to predict miRNA targets. We have limited our evaluation to target sites in 3′ UTRs. miRNAs directed at other types of targets or with dramatically different functions (e.g., in regulation of chromatin structure) might well use different rules. Accordingly, there may prove to be more targets than we can currently estimate. Further, there may be additional features, such as overall UTR context, that either enhance or limit the accessibility of predicted sites and hence their ability to function. For example, the rules about target site structure cannot explain the apparent requirement for the linker sequence observed in the *let-7/lin-41* regulation [48]. Further efforts toward experimental target site validation and systematic examination of UTR features can be expected to provide new insight into the function of miRNA target sites.

## Materials and Methods

### 

#### Fly strains

ptcGal4; EP miR278 was provided by Aurelio Teleman. The control, *hid, grim,* and *sickle* 3′ UTR reporter transgenes, and UAS-*miR-2b* are described in [19,26]. For UAS constructs for miRNA overexpression, genomic fragments including *miR-4* (together with *miR-286* and *miR-5*) and *miR-11* were amplified by PCR and cloned into UAS-DSred as described for UAS-*miR-7* [26]. Details are available on request. UAS-*miR-79* (also contains *miR-9b* and *miR-9c*) and UAS-*miR-6* (*miR-6–1, miR-6–2,* and *miR-6–3*) were kindly provided by Eric Lai. *dcr-1* ^Q1147X^ is described in [14].

#### Clonal analysis

Clones mutant for *dcr-1* ^Q1147X^ were induced in HS-Flp;dcr-1 FRT82/armadillo-lacZ FRT82 larvae by heat shock for 1 h at 38 °C at 50–60 h of development. Wandering third-instar larvae were dissected and labeled with rabbit anti-GFP (Torrey Pines Biolabs, Houston, Texas, United States; 1:400) and anti-β-Gal (rat polyclonal, 1:500).

#### Reporter constructs

The *bagpipe* 3′ UTR was PCR amplified from genomic DNA (using the following primers [enzyme sites in lower case]: AAtctaga AGGTTGGGAGTGACCATGTCTC and AActcgag TATTTAGCTCTCGGGTAGATACG) and cloned downstream of the tubulin promoter and EGFP (Clontech, Palo Alto, California, United States) in Casper4 as in [26].

#### Single target site constructs

Oligonucleotides containing the target site sequences shown in the figures were annealed and cloned downstream of tub>EGFP and upstream of SV40polyA (XbaI/XhoI). Clones were verified by DNA sequencing. Details are available on request.

#### EGFP intensity measurements

NIH image 1.63 was used to quantify intensity levels in miRNA-expressing and non-expressing cells from confocal images. Depending on the variation, between three and five individual discs were analyzed.

#### 3′ UTR alignments

For each D. melanogaster gene, we identified the D. pseudoobscura ortholog using TBlastn as described in [26]. We then aligned the D. melanogaster 3′ UTR obtained from the Berkeley Drosophila Genome Project to the D. pseudoobscura 3′ adjacent sequence (Human Genome Sequencing Center at Baylor College of Medicine) using AVID [49]. For individual examples, we manually mapped the D. melanogaster coding region to genomic sequence traces (National Center for Biotechnology Information trace archive) of *D. ananassae, D. virilis, D. simulans,* and D. yakuba by TBlastn and extended the sequences by Blastn-walking. These 3′ UTR sequences were then aligned to the D. melanogaster and D. pseudoobscura 3′ UTRs using AVID.

#### miRNA-sequences

Drosophila miRNA sequences were from [44,50,51] downloaded from Rfam (http://www.sanger.ac.uk/Software/Rfam/mirna/index.shtml). The 5′ non-redundant set (49 miRNAs) comprised *bantam, let-7, miR-1, miR-10, miR-11, miR-100, miR-124, miR-125, miR-12, miR-133, miR-13a, miR-14, miR-184, miR-210, miR-219, miR-263b, miR-275, miR-276b, miR-277, miR-278, miR-279, miR-281, miR-283, miR-285, miR-287, miR-288, miR-303, miR-304, miR-305, miR-307, miR-309, miR-310, miR-314, miR-315, miR-316, miR-317, miR-31a, miR-33, miR-34, miR-3, miR-4, miR-5, miR-79, miR-7, miR-87, miR-8, miR-92a, miR-9a,* and *miR-iab-4–5p.* Additional miRNAs in the 3′ non-redundant set were *miR-2b, miR-286, miR-306, miR-308, miR-311, miR-312, miR-313, miR-318,* and *miR-6.*

#### miRNA shuffles and mutants

For the completely shuffled miRNAs, we shuffled the miRNA sequence over the entire length and required all possible 8mer and 7mer seeds within the first nine bases to have an equal frequency (±15%) to the D. melanogaster 3′ UTRs (i.e., same single genome count). For the 3′ shuffled miRNAs, we shuffled the 3′ end starting at base 10 and required the shuffles to have equal (±15%) pairing energy to a perfect complement and to 10,000 randomly chosen sites. For each miRNA we created all possible 2-nt mutants (exchanging A to T or C, C to A or G, G to C or T, and T to A or G) within the seed (nucleotides 3–6) and chose the one with the closest alignment frequencies to the real miRNA in D. melanogaster 3′ UTRs and in the conserved sequences in D. melanogaster and D. pseudoobscura 3′ UTRs.

#### Seed matching and site evaluation

For each miRNA and seed type we found the 5′ match in the D. melanogaster 3′ UTRs and required it to be 100% conserved in an alignment to the D. pseudoobscura ortholog allowing for positional alignment errors of ±2 nt. When searching 7mer to 4mer seeds we masked all longer seeds to avoid identifying the same site more than once. For each matching site we extracted the 3′ adjacent sequence for both genomes, aligned it to the miRNA 3′ end starting at nucleotide 10 using RNAhybrid [35], and took the worse energy.

## Supporting Information

### Accession Numbers

The miRNA sequences discussed in this paper can be found in the miRNA Registry (http://www.sanger.ac.uk/Software/Rfam/mirna/index.shtml). NCBI RefSeq (http://www.ncbi.nlm.nih.gov/RefSeq/) accession numbers: *bagpipe* (NM_169958), *Brd* (NM_057541), *grim* (NM_079413), *hairy* (NM_079253), *hid* (NM_079412), *lin-14* (NM_077516), *lin-41* (NM_060087), and *Scr* (NM_206443). GenBank (http://www.ncbi.nlm.nih.gov/Genbank/) accession numbers: *sickle* (AF460844) and *D. simulans hairy* (AY055843).

## Abbreviations

*Brd*: *Bearded*
EGFP: enhanced green fluorescent protein
miRNA: microRNA
*Scr*: *Sex combs reduced*

## Abbreviations

Academic Editor: James C. Carrington: Oregon State University

## References

[pbio-0030085-b01] Ambros V (2004). The functions of animal microRNAs. Nature.

[pbio-0030085-b02] Lai EC (2003). microRNAs: Runts of the genome assert themselves. Curr Biol.

[pbio-0030085-b03] Carrington JC, Ambros V (2003). Role of microRNAs in plant and animal development. Science.

[pbio-0030085-b04] Bartel DP (2004). MicroRNAs: Genomics, biogenesis, mechanism, and function. Cell.

[pbio-0030085-b05] Pasquinelli AE, Reinhart BJ, Slack F, Martindale MQ, Kuroda MI (2000). Conservation of the sequence and temporal expression of let-7 heterochronic regulatory RNA. Nature.

[pbio-0030085-b06] Lim LP, Lau NC, Weinstein EG, Abdelhakim A, Yekta S (2003). The microRNAs of Caenorhabditis elegans. Genes Dev.

[pbio-0030085-b07] 7 Ambros V, Lee RC, Lavanway A, Williams PT, Jewell D (2003). MicroRNAs and other tiny endogenous RNAs in C. elegans. Curr Biol.

[pbio-0030085-b08] Grishok A, Pasquinelli AE, Conte D, Li N, Parrish S (2001). Genes and mechanisms related to RNA interference regulate expression of the small temporal RNAs that control C. elegans developmental timing. Cell.

[pbio-0030085-b09] Ketting RF, Fischer SE, Bernstein E, Sijen T, Hannon GJ (2001). Dicer functions in RNA interference and in synthesis of small RNA involved in developmental timing in C. elegans. Genes Dev.

[pbio-0030085-b10] Hutvagner G, McLachlan J, Pasquinelli AE, Balint E, Tuschl T (2001). A cellular function for the RNA-interference enzyme Dicer in the maturation of the let-7 small temporal RNA. Science.

[pbio-0030085-b11] Wienholds E, Koudijs MJ, van Eeden FJ, Cuppen E, Plasterk RH (2003). The microRNA-producing enzyme Dicer1 is essential for zebrafish development. Nat Genet.

[pbio-0030085-b12] Bernstein E, Kim SY, Carmell MA, Murchison EP, Alcorn H (2003). Dicer is essential for mouse development. Nat Genet.

[pbio-0030085-b13] Liu J, Carmell MA, Rivas FV, Marsden CG, Thomson JM (2004). Argonaute2 is the catalytic engine of mammalian RNAi. Science.

[pbio-0030085-b14] Lee YS, Nakahara K, Pham JW, Kim K, He Z (2004). Distinct roles for *Drosophila* Dicer-1 and Dicer-2 in the siRNA/miRNA silencing pathways. Cell.

[pbio-0030085-b15] Okamura K, Ishizuka A, Siomi H, Siomi MC (2004). Distinct roles for Argonaute proteins in small RNA-directed RNA cleavage pathways. Genes Dev.

[pbio-0030085-b16] Lee RC, Feinbaum RL, Ambros V (1993). The C. elegans heterochronic gene lin-4 encodes small RNAs with antisense complementarity to lin-14. Cell.

[pbio-0030085-b17] Reinhart BJ, Slack FJ, Basson M, Pasquinelli AE, Bettinger JC (2000). The 21-nucleotide let-7 RNA regulates developmental timing in Caenorhabditis elegans. Nature.

[pbio-0030085-b18] Johnston RJ, Hobert O (2003). A microRNA controlling left/right neuronal asymmetry in Caenorhabditis elegans. Nature.

[pbio-0030085-b19] Brennecke J, Hipfner DR, Stark A, Russell RB, Cohen SM (2003). *bantam* encodes a developmentally regulated microRNA that controls cell proliferation and regulates the pro-apoptotic gene hid in *Drosophila*. Cell.

[pbio-0030085-b20] Xu P, Vernooy SY, Guo M, Hay BA (2003). The *Drosophila* microRNA Mir-14 suppresses cell death and is required for normal fat metabolism. Curr Biol.

[pbio-0030085-b21] Chen CZ, Li L, Lodish HF, Bartel DP (2004). MicroRNAs modulate hematopoietic lineage differentiation. Science.

[pbio-0030085-b22] Poy MN, Eliasson L, Krutzfeldt J, Kuwajima S, Ma X (2004). A pancreatic islet-specific microRNA regulates insulin secretion. Nature.

[pbio-0030085-b23] Rhoades MW, Reinhart BJ, Lim LP, Burge CB, Bartel B (2002). Prediction of plant microRNA targets. Cell.

[pbio-0030085-b24] Jones-Rhoades MW, Bartel DP (2004). Computational identification of plant microRNAs and their targets, including a stress-induced miRNA. Mol Cell.

[pbio-0030085-b25] Bonnet E, Wuyts J, Rouze P, Van de Peer Y (2004). Evidence that microRNA precursors, unlike other non-coding RNAs, have lower folding free energies than random sequences. Bioinformatics.

[pbio-0030085-b26] Stark A, Brennecke J, Russell RB, Cohen SM (2003). Identification of Drosophila MicroRNA Targets. PLoS Biol.

[pbio-0030085-b27] Lewis BP, Shih IH, Jones-Rhoades MW, Bartel DP, Burge CB (2003). Prediction of mammalian microRNA targets. Cell.

[pbio-0030085-b28] Enright AJ, John B, Gaul U, Tuschl T, Sander C (2003). MicroRNA targets in *Drosophila*. Genome Biol.

[pbio-0030085-b29] Kiriakidou M, Nelson PT, Kouranov A, Fitziev P, Bouyioukos C (2004). A combined computational-experimental approach predicts human microRNA targets. Genes Dev.

[pbio-0030085-b30] Rajewsky N, Socci ND (2004). Computational identification of microRNA targets. Dev Biol.

[pbio-0030085-b31] Lai EC (2004). Predicting and validating microRNA targets. Genome Biol.

[pbio-0030085-b32] Lai EC (2002). Micro RNAs are complementary to 3′ UTR sequence motifs that mediate negative post-transcriptional regulation. Nat Genet.

[pbio-0030085-b33] Saxena S, Jonsson ZO, Dutta A (2003). Small RNAs with imperfect match to endogenous mRNA repress translation. Implications for off-target activity of small inhibitory RNA in mammalian cells. J Biol Chem.

[pbio-0030085-b34] Doench JG, Sharp PA (2004). Specificity of microRNA target selection in translational repression. Genes Dev.

[pbio-0030085-b35] Rehmsmeier M, Steffen P, Hochsmann M, Giegerich R (2004). Fast and effective prediction of microRNA/target duplexes. RNA.

[pbio-0030085-b36] Wightman B, Ha I, Ruvkun G (1993). Posttranscriptional regulation of the heterochronic gene lin-14 by lin-4 mediates temporal pattern formation in C. elegans. Cell.

[pbio-0030085-b37] Ha I, Wightman B, Ruvkun G (1996). A bulged lin-4/lin-14 RNA duplex is sufficient for Caenorhabditis elegans lin-14 temporal gradient formation. Genes Dev.

[pbio-0030085-b38] Abrahante JE, Daul AL, Li M, Volk ML, Tennessen JM (2003). The Caenorhabditis elegans hunchback-like gene lin-57/hbl-1 controls developmental time and is regulated by microRNAs. Dev Cell.

[pbio-0030085-b39] Lin SY, Johnson SM, Abraham M, Vella MC, Pasquinelli A (2003). The C. elegans hunchback homolog, hbl-1, controls temporal patterning and is a probable microRNA target. Dev Cell.

[pbio-0030085-b40] Lai EC, Posakony JW (1997). The Bearded box, a novel 3′ UTR sequence motif, mediates negative post-transcriptional regulation of Bearded and Enhancer of split Complex gene expression. Development.

[pbio-0030085-b41] Yekta S, Shih IH, Bartel DP (2004). MicroRNA-directed cleavage of HOXB8 mRNA. Science.

[pbio-0030085-b42] Doench JG, Petersen CP, Sharp PA (2003). siRNAs can function as miRNAs. Genes Dev.

[pbio-0030085-b43] John B, Enright AJ, Aravin A, Tuschl T, Sander C (2004). Human microRNA targets. PLoS Biol.

[pbio-0030085-b44] Lai EC, Tomancak P, Williams RW, Rubin GM (2003). Computational identification of *Drosophila* microRNA genes. Genome Biol.

[pbio-0030085-b45] Slack FJ, Basson M, Liu Z, Ambros V, Horvitz HR (2000). The lin-41 RBCC gene acts in the C. elegans heterochronic pathway between the let-7 regulatory RNA and the LIN-29 transcription factor. Mol Cell.

[pbio-0030085-b46] Bartel DP, Chen CZ (2004). Micromanagers of gene expression: The potentially widespread influence of metazoan microRNAs. Nat Rev Genet.

[pbio-0030085-b47] Hobert O (2004). Common logic of transcription factor and microRNA action. Trends Biochem Sci.

[pbio-0030085-b48] Vella MC, Choi EY, Lin SY, Reinert K, Slack FJ (2004). The C. elegans microRNA let-7 binds to imperfect let-7 complementary sites from the lin-41 3′UTR. Genes Dev.

[pbio-0030085-b49] Bray N, Dubchak I, Pachter L (2003). AVID: A global alignment program. Genome Res.

[pbio-0030085-b50] Lagos-Quintana M, Rauhut R, Lendeckel W, Tuschl T (2001). Identification of novel genes coding for small expressed RNAs. Science.

[pbio-0030085-b51] Aravin AA, Lagos-Quintana M, Yalcin A, Zavolan M, Marks D (2003). The small RNA profile during Drosophila melanogaster development. Dev Cell.

